# Bcl-xL inhibition - a novel strategy for glioma therapy

**DOI:** 10.18632/aging.101074

**Published:** 2016-09-28

**Authors:** Georg Karpel-Massler, Markus D. Siegelin

**Affiliations:** Department of Pathology and Cell Biology, Columbia University Medical Center, New York, NY 10032, USA

**Keywords:** L-asparaginase, ABT263, glioblastoma, apoptosis, Usp9X

Malignant gliomas represent highly aggressive brain tumors and include glioblastoma as the most common primary brain tumor in adults. This disease is related to a life expectancy of less than 2 years after diagnosis despite best standard of care involving surgery, chemo- and radiotherapy. With a relatively modest, but significant effect on patient survival, the alkylating agent temozolomide is commonly administered to patients with glioblastoma, however, even when combined with radiotherapy the prognosis of patients suffering from a glioblastoma does not improve in a satisfactory manner [1]. Thus, there is an urgent quest for unraveling more efficient therapeutic approaches. Amongst the several routes scientists and clinicians have taken is the strategy to interfere with amino acid metabolism. In our recently published manuscript, we have taken advantage of this approach by utilizing L-asparaginase, which is an approved drug for acute lymphoblastic leukemia, for the treatment of glio-blastoma. While this idea has been pursued for brain tumors before, we have further refined this strategy by combining L-asparaginase with an orally available inhibitor targeting Bcl-xL [2]. In that regard, we utilized one of the recently designed BH3-mimetics, ABT-263, which aside from Bcl-xL also interferes with Bcl-2 [3]. While other compounds that interfere with Bcl-xL and or Bcl-2 have been described before, the ABT-compounds are unique by their high-affinity target binding within the low nanomolar range. Recently, in a study led by researchers from the University of Ulm, a structural relative of ABT-263 (ABT-199, Venetoclax) demonstrated remarkable efficacy in chronic lympho-cytic leukemia patients that harbor a loss of chromosome 17p [4]. These results led to the designation of “fast-track approval” for ABT-199 (Venetoclax) by the US Food and Drug Administration [5].

In our study, we demonstrated that the combination treatment of ABT-263 and L-asparaginase resulted in a potent synergistic reduction in proliferation of a range of genetically diverse glioblastoma cells [2]. This was also true in glioblastoma cells that were resistant to L-asparaginase alone. Mechanistically, the combination treatment induced apoptosis with activation of effector caspases and loss of mitochondrial membrane potential. It remains to be determined as to whether other forms of cell death are involved. For instance, it is known that L-asparaginase induces autophagy in model systems of myeloid leukemia. While a significant proportion of glioblastomas harbor mutations in *TP53*, it appeared that the combination treatment does not require an active p53 pathway since mutated p53 cells responded to ABT-263 and L-asparaginase. On the molecular level, we found that L-asparaginase regulates the levels of the deubiquitinase Usp9X, an enzyme found to be up-regulated in malignant glial tumors, and another anti-apoptotic Bcl-2 family member, Mcl-1 [2]. While the ABT-compounds efficiently target Bcl-xL and Bcl-2, they do not tightly interact with Mcl-1. This comes at a price since BIM and BAK, two pro-apoptotic Bcl-2 family members, will be redirected to Mcl-1 upon Bcl-xL inhibition. In turn, Mcl-1 sequesters these proteins to interfere with BAX/BAK activation and permeabiliza-tion of the outer mitochondrial membrane. Therefore, depletion of Mcl-1 by L-asparaginase will mitigate this effect and efficiently prime tumor cells to apoptosis induction by ABT-263. L-asparaginase-mediated reduction in Mcl-1 levels most likely occurs at multiple levels. Given that 1) Usp9X interacts and de-ubiquitinates Mcl-1 [6] and 2) L-asparaginase depletes Usp9X levels [2], it appears likely that L-asparaginase regulates Mcl-1 levels in part by regulating its stability. Since Mcl-1 has a short half-life an alternative mechanism by which L-asparaginase might regulate Mcl-1 levels is through interference with protein synthesis and mTORC1 signaling [7]. Other than regulating Usp9X and Mcl-1, L-asparaginase also modulated the expression of the pro-apoptotic molecule Noxa in p53 mutated glioblastoma cells. Noxa is known to inhibit the function of Mcl-1 through several mechanisms involving direct binding to Mcl-1. We found that knockdown of Noxa protected from ABT263 and L-asparaginase-mediated cell death in glioblastoma cells. How L-asparaginase up-regulates Noxa in our setting remains to be determined. While Noxa is regulated by p53, other factors have been identified, such as the Activating Transcription Factor 4 (ATF4). Since L-asparaginase interferes with protein synthesis, it appears likely that L-asparaginase up-regulates protein levels of ATF4 via enhanced phosphorylation of eif2α. In turn, ATF4 would increase transcription of Noxa which is consistent with our findings since L-asparaginase increased both the Noxa transcript and protein in glioblastoma cells. Finally, we verified whether the combination of ABT-263 and L-asparaginase is suitable for *in vivo* treatment. For that, we utilized a proneural model of glioma and showed that animals, receiving both ABT-263 and L-asparagi-nase, displayed a significantly decreased tumor growth rate compared to control or single treatments. No significant toxicities were noted during or after the treatment. These observations serve as a proof of principle for this treatment strategy as potentially feasible, effective and to be further explored in the setting of clinical trials.
